# Validity of AI-Driven Markerless Motion Capture for Spatiotemporal Gait Analysis in Stroke Survivors

**DOI:** 10.3390/s25175315

**Published:** 2025-08-27

**Authors:** Balsam J. Alammari, Brandon Schoenwether, Zachary Ripic, Neva Kirk-Sanchez, Moataz Eltoukhy, Lauri Bishop

**Affiliations:** 1 Department of Physical Therapy, Miller School of Medicine, University of Miami, Coral Gables, FL 33146, USA; bja81@miami.edu (B.J.A.); nkirksanchez@miami.edu (N.K.-S.); meltoukhy@miami.edu (M.E.); 2 Department of Kinesiology and Sport Sciences, School of Education & Human Development, University of Miami, Coral Gables, FL 33146, USA; bxs1256@miami.edu (B.S.); zar17@miami.edu (Z.R.); 3 Department of Orthopaedics, Miller School of Medicine, University of Miami, Coral Gables, FL 33136, USA

**Keywords:** stroke, artificial intelligence, motion capture, biomechanics, gait, spatiotemporal parameters, markerless motion capture

## Abstract

**Highlights:**

**What are the main findings?**

- KinaTrax (HumanVersion 8.2, KinaTrax Inc., Boca Raton, FL, USA) markerless motion capture is a valid system for measuring spatiotemporal gait metrics after stroke during comfortable and fast walking speeds.
- Measures of stride width and single-limb support time should be interpreted with caution.

**What is the implication of the main finding?**

- KinaTrax is a promising sensor-free and streamlined gait analysis technology that can be integrated into gait rehabilitation after stroke.

**Abstract:**

Gait recovery after stroke is a primary goal of rehabilitation, therefore it is imperative to develop technologies that accurately identify gait impairments after stroke. Markerless motion capture (MMC) is an emerging technology that has been validated in healthy individuals. Our study aims to evaluate the validity of MMC against an instrumented walkway system (IWS) commonly used to evaluate gait in stroke survivors. Nineteen participants performed three comfortable speed (CS) and three fastest speed (FS) walking trials simultaneously recorded with IWS and MMC system, KinaTrax (HumanVersion 8.2, KinaTrax Inc., Boca Raton, FL, USA). Pearson’s correlation coefficient and intraclass correlation coefficient (ICC (3,1), 95%CI) were used to evaluate the agreement and consistency between systems. Furthermore, Bland–Altman plots were used to estimate bias and Limits of Agreement (LoA). For both CS and FS, agreements between MMC and IWS were good to excellent in all parameters except for non-paretic single-limb support time (SLS), which revealed moderate agreement during CS. Additionally, stride width and paretic SLS showed poor agreement in both conditions. Biases eliminated systematic errors, with variable LoAs in all parameters during both conditions. Findings indicated high validity of MMC in measuring spatiotemporal gait parameters in stroke survivors. Further validity work is warranted.

## 1. Introduction

Stroke is the second leading cause of death and the third leading cause of disability worldwide [1]. While medical advances have improved survival rates from stroke [2], increased survival rates result in more individuals living with long-term disability. More than half of stroke survivors lose their ability to walk independently, and over 40% present with lower extremity motor impairments and gait deficits [3,4]. Post-stroke gait deficits are characterized by deviations in spatiotemporal gait parameters that include, but are not limited to, reduced walking speed, decreased time in the stance phase of gait, and large variability in stride length [5,6,7]. The atypical gait patterns often adopted by stroke survivors lead to increases in ambulatory energy costs, reduced endurance in gait-related activities, and overall decreased community participation as compared to peers without stroke [8,9,10]. Therefore, improvement in gait function is a top rehabilitation priority of stroke survivors [11]. Rehabilitation and biomechanical scientists have collaborated to design gait analysis technologies aimed to improve the functionality in the population of stroke survivors. Particularly, advanced gait analysis techniques provide individualized diagnosis and treatment of post-stroke gait impairments, facilitating the efforts of rehabilitation providers to guide clinical decision-making, predict recovery, and track rehabilitation progress [12,13,14,15].

Unlike technology-free methods, such as the 10 m walk test (10MWT) or six min walk test, technology-driven gait analysis systems enable objective estimation of joint motion and spatiotemporal gait measures using automated data processing techniques [16,17]. For example, the instrumented walkway system (IWS) has become an industry standard for measuring spatiotemporal gait metrics [18,19]. IWS has gained popularity in clinical gait analysis due to its user-friendly operation, low cost of equipment, and minimal training requirement [16]. Although IWS provides valid information on spatiotemporal gait characteristics, it has several critical limitations [16]. First, IWS is not designed to measure additional biomechanical gait features such as kinematics and kinetics, limiting three-dimensional (3D) body movements to two-dimensional (2D) representations for further analyses. Second, spatiotemporal measures are restricted to full steps on a pre-sized mat, which can limit the number of strides available for accurate gait analysis. Third, IWS lacks the flexibility to assess gait patterns other than straight walking, which is rarely performed solely in real life, where navigation, turning, and multidirectional walking are inherent requirements of walking. Fourth and most notably, IWS gait analysis is limited to an artificial laboratory or clinical environment, where no obstacles or other individuals are allowed during gait analysis [16]. These environmental limitations in IWS can change movement and constrain gait patterns that stroke survivors may adopt in everyday life. Consequently, the outcomes of IWS might not be entirely representative of post-stroke gait outside the lab within environmental contexts that are salient to the stroke survivor. The same concern about the ecological validity of gait analysis also applies to other standardized technologies [20].

In an effort to more accurately characterize natural movements in home and community settings, artificial intelligence-driven markerless motion capture (MMC) has rapidly emerged as an alternative to standardized technology-based gait analysis [20,21]. MMC performs sensor-free gait analysis by estimating human poses using Convolutional Neural Networks (CNNs) or closely related deep learning techniques [22]. This approach has allowed MMC to perform 3D gait analysis with minimal restrictions on distance or environmental context. Prior work has established the validity and reliability of vision-based MMC using single, dual, and multi-view approaches [23,24,25,26,27]. Specifically, consumer-grade MMC systems like OpenPose, Kinect, or SMARTGAIT allow user-friendly and customizable gait analysis, with real-time data analysis that could be easily integrated in clinical settings [23,24,25,26,27]. However, these systems have a limited ability to produce highly precise gait-related outcomes. Alternatively, research-based MMC systems, like Thiea 3D, have been introduced as more precise MMC systems, and have been validated for spatiotemporal gait analysis mainly in healthy individuals [28], and in non-targeted clinical populations [29]. Yet to date, very little is known about the validity of precise multi-view 3D MMC systems in individuals with stroke.

The KinaTrax MMC system was originally developed for in-game biomechanics assessment of baseball pitching and hitting. The KinaTrax MMC system within the laboratory comprises eight high speed digital video cameras (Kowa, Kowa Optimed, Duesseldorf, Germany) that capture synchronously at 100 Hz. The camera system is set up to maximize a region of interest approximately 5 square meters from floor to ceiling. Video data are collected manually within an internally housed machine and then processed using a standard analysis framework. This includes calibration through background alignment using a reference image taken prior to the collection of motion trials to account for any camera drift, however unlikely this may be, and configure the virtual laboratory. The reference image is shifted to match a standardized area at the center of the region of interest. This is followed by methods previously described (by Ripic et al. [30] including video aggregation from all eight cameras on the local machine before the CNN is implemented using a bounding box detection algorithm and additional layers of 2D to 3D keypoint estimations. The final result is triangulated 3D coordinates from the virtual model that represents each of the 40 keypoints using a 4 × 4 transformation matrix. This allows for the scaling of a standard biomechanical model in the industry standard file configuration .C3D.

Recently, KinaTrax Inc., Boca Raton, FL, USA, developed an alternative to their baseball-specific pose estimation models in the form of a general human model. This has been examined for validity and reliability when assessing spatiotemporal gait parameters and kinematic waveforms in healthy adults [31,32] and adults with Parkinson’s Disease [30] in gait. However, the validity of the KinaTrax to measure spatiotemporal gait characteristics in stroke survivors is still unknown. The aim of this study is to evaluate the concurrent validity of spatiotemporal gait parameters obtained from the KinaTrax MMC compared to the rehabilitation industry standard of IWS in stroke survivors. We hypothesize that there will be a good to excellent agreement in spatiotemporal gait parameters measured by the KinaTrax MMC and the IWS among individuals post-stroke.

## 2. Materials and Methods

### 2.1. Study Cohort

A convenience sample of nineteen stroke survivors were recruited from the UHealth Clinic at University of Miami and Jackson Health System, Miami, Florida, USA, between February 2024 to April 2025. Patients were included if they were 18–75 years-old, more than one-month post-stroke, home/community-dwelling (not in an inpatient rehabilitation unit or sub-acute nursing facility), and able to walk independently with or without an assistive device (Functional Ambulation Category (FAC) ≥ 3). Exclusion criteria included a diagnosis of cerebellar stroke, presence of neurodegenerative disease (e.g., Parkinson’s Disease, multiple sclerosis), or having a musculoskeletal, cardiovascular, or chronic disease with a severity that would significantly impact mobility. Also, individuals with hearing or visual defects that limited mobility, and those who were unable to follow three-step commands were excluded from participation. The study was approved by the Institutional Review Boards of the University of Miami and Jackson Health System. Consent documents were reviewed, and participants had the opportunity to ask any study-related questions. After which, written informed consent was obtained from each participant prior to initiation of study procedures.

### 2.2. Study Procedures

A cross–sectional study design was used. Prior to arrival at the study location, participants were instructed to wear their preferred ‘street’ attire provided they refrained from baggy or otherwise bulking clothing that could complicate pose estimation due to obscure body shapes, this included the typical footwear such as tennis shoes or sneakers. At the participants’ arrival, the Montreal Cognitive Assessment (MoCA) was used to screen cognition, and the National Institute of Health Stroke Scale (NIHSS) characterized stroke severity. FAC scale measures were also recorded.

A 4.3 m IWS (Protokinetics ZenoTM Walkway Gait Analysis System, Havertown, PA, USA) was placed in the center of a 10 m walkway course in a laboratory equipped with eight cameras of the KinaTrax (KinaTrax Inc., Boca Raton, FL, USA) MMC system. The location of the IWS was pre–measured to ensure that both the IWS and the MMC captured at least one complete gait cycle simultaneously. The IWS was calibrated prior to each data collection for each participant using baseline scanning. The MMC was calibrated using a static reference image rendered during post-processing. Data from IWS was synced with MMC in post–processing.

**Figure 1:**
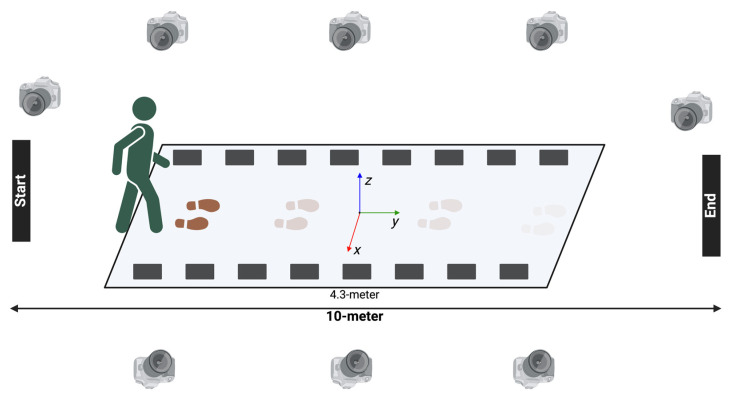
Experimental schema showing a participant walking during a 10-m walk test inside the instrumented walkway system with simultaneous recording from eight KinaTrax markerless motion capture cameras.

The 10MWT was performed over the IWS with simultaneous recording via the MMC (Figure 1). Participants were first instructed to walk as comfortably and as safely as possible (comfortable speed, (CS)). A practice trial was conducted prior to initiating CS 10MWT. Measures were recorded in a series of three trials. The 10MWT was then repeated for three additional trials at a fast speed in which the participants were asked to “walk as fast and as safely as possible” (fastest speed (FS)). Trials that included instances in which participants walked outside the IWS were repeated.

### 2.3. Data Analysis

Data from the IWS were analyzed at a 120 Hz sampling rate. No down filter was used. The ProtoKinetics Movement Analysis Software ((PKMAS, Version 5.08C2i4, ProtoKinetics, Havertown, PA, USA) detected gait events (i.e., heel-strike and toe-off) using the sense of foot pressure and automatically calculated spatiotemporal parameters. Each trial was then manually processed to exclude incomplete steps and gait cycles that were not captured by the MMC, before finally exporting the data of one gait cycle. The following spatiotemporal parameters were included in our analysis: gait speed, double–limb support time (DLS), stride length, stride width, single–limb support time (SLS), stance time, and step length. Data from the KinaTrax MMC system were collected at a sampling rate of 100 Hz, an immutable setting of the system, then processed using a proprietary pipeline. This is performed by incorporating the static reference image to ensure the camera region of interest is consistent from session to session. Following this, data were transferred to a secure cloud server for biomechanical post–processing using a standard biomechanics modeling software (Visual3D, Version 2023, HAS–Motion, Kingston, ON, Canada). The modeling software operates with a scripting syntax used to create custom analysis pipelines. These allow for flexibility when handling data from multiple tracked individuals, including the participant and surrounding therapists present to ensure participant safety. The MMC processing started with scaling a 15–segment biomechanical model, assuming 6 degrees of freedom across each segment. This was followed by identifying the participant using the average mediolateral (side-to-side) position for the trial. Aiding therapists only occupied the edge of the region of interest, making it simple to delineate the participant from the therapists. To determine the direction of gait progression, the timing of the maximum and minimum anteroposterior (forward–backward) position in the laboratory coordinate system was calculated and evaluated based on the order of occurrence. Data were then filtered using a low pass butterworth filter with a cutoff frequency of 10 Hz [33] and the inverse kinematics optimization was performed. Using the filtered data, gait events were detected for heel-strike (HS) and toe–off (TO) events using the velocity-based method of Zeni et al. [34]. Following gait event detection, gait cycle selection was performed, and spatiotemporal parameters were calculated. For each individual participant, three trials per condition were included. In incidents where one trial was not captured by both systems due to unreconcilable errors discovered in post-processing, the trial was discarded and two trials per condition were included. The gait cycle in the IWS was synchronized with the gait cycle in the MMC using timestamps and the direction of movement for each gait cycle. Within each trial, one complete gait cycle was analyzed.

### 2.4. Statistical Analysis

Histograms and the Shapiro–Wilk tests were used to evaluate the normality of the data. Descriptive data were generated for all variables using median (IQR) for continuous variables, and n (%) for categorical variables. Validity was assessed with a correlation analysis using the Pearson correlation coefficient (r). Absolute agreement and relative consistency between the MMC and IWS were also analyzed, using ICC estimates with 95% confidence intervals calculated based on a mean rating (k = 3), single measure, 2–way mixed–effects model. Although the three tests indicate agreement, each represents a different assumption, or rather, a different form of agreement [35]. The correlation assesses linear changes in the relationship between two measurements, while the absolute agreement assesses their identical contrasts, and the relative consistency assesses if the two measures vary similarly, without necessarily assuming linearity [35]. Therefore, we included the three measures to estimate the validity of the MMC holistically. For interpretation, correlation coefficients and ICCs < 0.5 were considered poor, 0.5–0.75, moderate, 0.75–0.9, good, and >0.9 indicated excellent agreement between the systems [36]. Finally, Bland–Altman plots were created for each gait parameter to estimate the bias (mean difference) and Limits of Agreement (LoA). A priori, an alpha level of <0.05 was considered significant. Statistical analysis was performed using IBM SPSS Statistics for macOS, Version 28.0.1.1 (14), Armonk, NY, USA: IBM Corp.

## 3. Results

### 3.1. Descriptive Statistics

A total of 19 participants with 54 CS trials and 51 FS trials were included in the analysis. Participants’ demographics and stroke-related characteristics are shown in Table 1. The sample included individuals with a median (IQR) age of 63 (55–65) years old; 53% male; 21% Non-Hispanic Whites, 37% Non-Hispanic Blacks, and 42% Hispanics. Median (IQR) time since stroke was 33 (14–109) months, and nearly half of the participants (53%) were right-sided hemiparetic. According to the NIHSS, 14 (74%) participants had mild stroke (NIHSS 0–5), and 5 (26%) presented with moderate stroke (NIHSS 6–15) [37]. Within our participants, 2 (11%) had an FAC score of 3, 8 (42%) scored 4, and 9 (47%) scored 5. Cognition, as screened by the MoCA, had a median (IQR) of 23 (20–25).

### 3.2. Two-Limb Parameters

**Figure 2:**
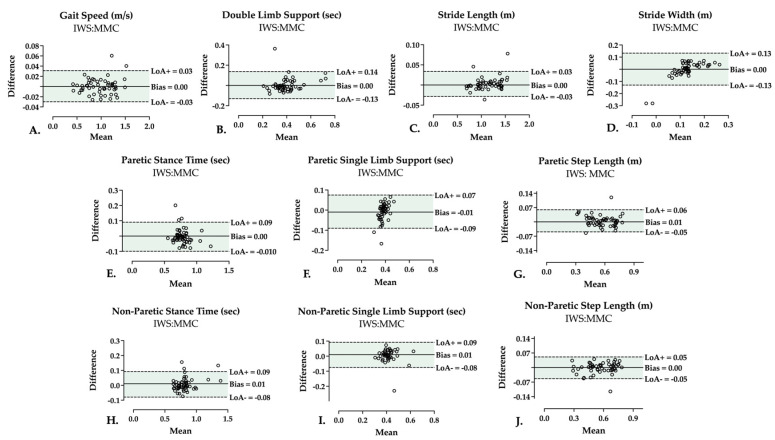
Comfortable speed Bland–Altman plots showing bias (mean difference), upper Limit of Agreement (LoA+), and lower Limit of Agreement (LoA−) of the two-limb parameters (**A**–**D**), and single-limb parameters of each paretic (**E**–**G**) and non-paretic (**H**–**J**) leg. Individual points represent each included trial. Area of agreement is highlighted.

A summary of agreement indicators (correlation coefficients, ICCs (agreement), ICCs (consistency)) between MMC and IWS in measuring spatiotemporal parameters during CS is presented in Table 2. Correlation coefficients were excellent for gait speed [r, 0.996] and stride length [r, 0.997], good for DLS [r, 0.768], and poor for stride width [r, 0.554]. ICC for absolute agreement was excellent for gait speed [ICC (3,1) 0.999, (0.997–0.999)] and stride length [ICC (3,1) 0.998, (0.997–0.999)], and good for DLS [ICC (3,1) 0.804, (0.684–0.881)]. Stride width had poor absolute agreement [ICC (3,1) 0.442, (0.198–0.634)] between the two systems. Similarly, ICC for relative consistency was excellent for gait speed [ICC (3,1) 0.998 (0.997–0.999)] and stride length [ICC (3,1) 0.998 (0.997–0.999)], and good for DLS [ICC (3,1) 0.801 (0.680–0.880)]. Relative consistency was poor for stride width [ICC (3,1) 0.438 (0.195–0.630)]. Bland–Altman plots with bias and LoA during CS are shown in Figure 2. Bias (LoA) was 0.00 (−0.03 to +0.03) m/s for gait speed, 0.00 (−0.13 to +0.14) s for DLS, 0.00 (−0.03 to +0.03) m for stride length, and 0.00 (−0.13 to +0.13) m for stride width.

**Figure 3:**
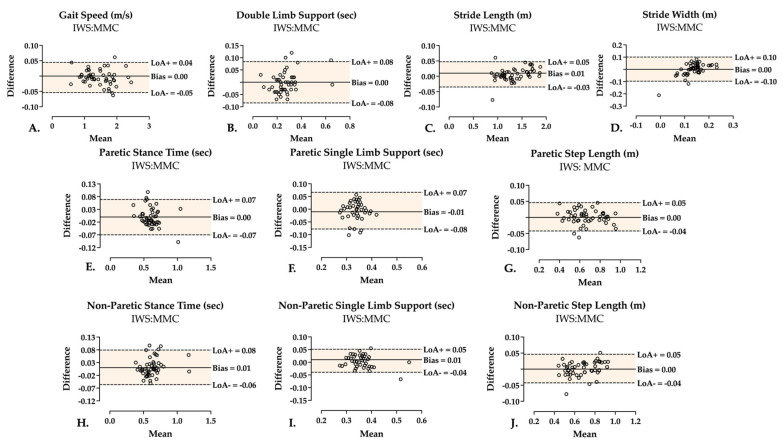
Fastest speed Bland–Altman plots showing bias (mean difference), upper Limit of Agreement (LoA+), and lower Limit of Agreement (LoA−) of the two-limb parameters (**A**–**D**) and single-limb parameters of each paretic (**E**–**G**), and non-paretic (**H**–**J**) leg. Individual points represent each included trial. Area of agreement is highlighted.

Similar to CS, FS two-limb parameters showed excellent correlation for gait speed [r, 0.996] and stride length [r, 0.997], good correlation for DLS [r, 0.843], and poor correlation for stride width [r, 0.508]. Absolute agreement was excellent for gait speed [ICC (3,1) 0.998 (0.997–0.999)] and stride length [ICC (3,1) 0.997 (0.995–0.998)], good for DLS [ICC (3,1) 0.917 (0.859–0.952)], and was poor for stride width [ICC (3,1) 0.476 (0.230–0.663)]. Likewise, relative consistency for gait speed, DLS, stride length, and stride width were [ICC (3,1) 0.998 (0.997–0.999)], [ICC (3,1) 0.916 (0.857–0.951)], [ICC (3,1) 0.997 (0.995–0.998)], and [ICC (3,1) 0.471 (0.227–0.659)], respectively, indicating good to excellent consistency in all the two-limb parameters except stride width during FS. Detailed FS results are reported in Table 3. Regarding bias and LoA during FS, Figure 3 illustrates Bland–Altman plots with bias and LoA during FS. Gait speed bias (LoA) was 0.00 (−0.05 to 0.04) m/s, DLS was 0.00 (−0.08 to 0.08) s, stride length was 0.01 (−0.03 to 0.05) m, and stride width was 0.00 (−0.10 to 0.10) m.

### 3.3. Single-Limb Parameters

Regarding CS single-limb parameters (Table 2), correlation was good for paretic [r, 0.858] and non-paretic [r, 0.867] stance time, and excellent for paretic [r, 0.973] and non-paretic [r, 0.969] step length. Correlation coefficients revealed a poor correlation [r, 0.431] in paretic SLS, while it showed a moderate correlation [r, 0.691] in non-paretic SLS. ICC for absolute agreement demonstrated good agreement in paretic stance time [ICC (3,1) 0.912 (0.854–0.948)], and excellent agreement in each non-paretic stance time [ICC (3,1) 0.958 (0.928–0.975)], paretic step length [ICC (3,1) 0.976 (0.959–0.986)] and non-paretic step length [ICC (3,1) 0.982 (0.968–0.989)]. ICCs for SLS indicated a poor agreement in measuring SLS in the paretic limb [ICC (3,1) 0.314 (0.057–0.533)], and a moderate agreement in SLS of the non-paretic limb [ICC (3,1) 0.731 (0.578–0.834)]. Similar findings are demonstrated in relative consistency, where stance time showed good consistency in the paretic limb [ICC (3,1) 0.911 (0.852–0.948)], and excellent consistency in the non-paretic limb [ICC (3,1) 0.958 (0.928–0.975)]. Excellent consistency was also revealed in step length of both paretic [ICC (3,1) 0.976 (0.959–0.986)] and non-paretic [ICC (3,1) 0.981 (0.968–0.989)] limbs. Consistency was poor for paretic SLS [ICC (3,1) 0.317 (0.057–0.538)] and moderate for non-paretic SLS [ICC (3,1) 0.734 (0.582–0.837)]. In terms of bias and LoA, stance time bias (LoA) was 0.00 (−0.10 to 0.09) s SLS was −0.01 (−0.09 to 0.07) s, and step length was 0.01 (−0.05 to 0.06) m. Bias (LoA) in the non-paretic side was 0.01 (−0.08 to 0.09) s for both stance time and SLS, and was 0.00 (−0.05 to 0.05) m for step length (Figure 2).

With respect to single–limb parameters during FS (Table 3), correlation was good for paretic [r, 0.928] and non–paretic [r, 0.942] stance time, and was excellent for paretic [r, 0.985] and non–paretic [r, 0.977] step length. SLS revealed a poor correlation in measures of paretic SLS [r, 0.446], and good correlation in non-paretic SLS [r, 0.846]. ICC of absolute agreement demonstrated excellent agreement for paretic [ICC (3,1) 0.960 (0.931–0.977)] and non-paretic [ICC (3,1) 0.969 (0.944–0.983)] stance time, and for paretic [ICC (3,1) 0.989 (0.981–0.994)] and non-paretic [ICC (3,1) 0.987 (0.978–0.993)] step length. Paretic SLS showed poor agreement [ICC (3,1) 0.480 (0.240–0.665)], and non-paretic SLS revealed a good agreement [ICC (3,1) 0.886 (0.807–0.934)] between the systems. Similarly, relative consistency was excellent for paretic [ICC (3,1) 0.959 (0.929–0.976)] and non-paretic [ICC (3,1) 0.972 (0.951–0.984)] stance time, and for paretic [ICC (3,1) 0.989 (0.980–0.994)] and non–paretic [ICC (3,1) 0.987 (0.977–0.993)] step length. SLS consistency was poor in the paretic limb [ICC (3,1) 0.481 (0.239–0.667)] and good in the non–paretic limb [ICC (3,1) 0.890 (0.815–0.936)]. During FS, bias (LoA) in the paretic leg was 0.00 (−0.07 to 0.07) s for stance time, −0.01 (−0.08 to 0.07) s for SLS, and 0.00 (−0.04 to 0.05) m for step length. Non-paretic leg bias (LoA) for stance time, SLS, and step length was 0.01 (−0.06 to 0.08) s, 0.01 (−0.04 to 0.05) s, and 0.00 (−0.04 to 0.05) m, respectively (Figure 3).

## 4. Discussion

The aim of this study was to test the concurrent validity (correlation, absolute agreement, relative consistency, and bias (mean difference) with LoA) of spatiotemporal gait parameters obtained simultaneously from the KinaTrax MMC against a standard IWS.

We found that MMC has good to excellent validity in measuring gait speed, stride length, stance time (paretic and non–paretic), and step length (paretic and non–paretic). Our findings are similar to those reported in studies comparing MMC with IWS [28,29,38,39]. Particularly, studies that compared both consumer–grade MMC [38,39] and research-based MMC [28,29] to IWS in individuals with stroke reported excellent agreement in all the above–mentioned parameters. Our findings are also consistent with prior work that validated the KinaTrax in healthy adults and adults with Parkinson’s [30]. Although DLS had a good agreement in our findings, similar to McGuirk et al. [29], the lower range of confidence brings into question the actual level of the DLS agreement between the systems. Our DLS observation is also consistent with Kanko et al. [28], who found lower DLS agreement between MMC and IWS as compared to the other two–limb spatiotemporal gait metrics in a population without stroke. Conversely, Lonini et al. [38] reported higher agreement in DLS, although this might be due to the customized nature of their Human Pose Estimation (HPE) method tailored for individuals post–stroke. Additionally, Lonini et al. [38] included only eight participants with chronic stroke, which may have biased the study sample toward fewer gait deficits than our sample, leading to more accurate measurement in DLS. In fact, our sample included stroke survivors within a wide range of stroke stages, including the sub–acute stage. Thus, some of our study cohort presented with moderate gait deficits that might resolve in chronic stages of stroke [40].

Notably, we showed a poor agreement between MMC and IWS in estimating stride width. Prior work comparing MMC with marker–based motion capture in a population of stroke yielded a similarly poor agreement in step width, which would compromise measures of stride width [41,42]. A plausible reason for the low agreement in stride width is the limited walkway space, which is known to limit the IWS spatial resolution and therefore reduce the reliability of base of support measures recorded by the IWS [28,43]. Another plausible explanation is the inherent stride-to-stride variability in individuals with stroke, which may limit MMC’s ability to identify virtual keypoints in the foot, regardless of the reference system used [5,6,7]. Although studies have suggested including a minimum of ten strides in gait analysis to overcome this variability [44,45], it is unlikely that the inclusion of one gait cycle in our analysis was the reason for the poor ICC in stride width, as other studies with similar findings did include a minimum of ten stride width measurements in their analysis [28]. In contrast to our findings, prior KinaTrax validation work by Ripic et al. [30] indicated excellent agreement in stride width compared to marker-based motion capture, and McGuirk et al. [29] demonstrated good agreement of stride width compared to IWS. Nonetheless, Ripic et al. [30] work did not include individuals with stroke, and McGuirk et al. [29] sample only included three stroke survivors. Thus, it is difficult to infer the independent effect of stroke gait variability on the MMC validity in these two studies.

With respect to single–limb parameters, our findings indicated poor agreement in measuring paretic SLS and moderate agreement in measuring non–paretic SLS between MMC and IWS. The difference in agreement level between paretic and non–paretic SLS is not surprising. Given the hemiparetic nature of stroke and the associated motor-related impairments [46], we expect the reliability of gait analysis to be lower in the paretic leg. More specifically, the poor agreement in paretic SLS might be due to the presence of foot drop in some of our study cohort. HS and TO events were determined using the decrease or increase in forward velocity of the respective keypoints on the KinaTrax MMC general human motion model. Therefore, tracking errors caused by foot dragging may misrepresent the precise timing of HS or TO gait events. Particularly, given the flat HS occurring during foot drop, MMC gait event detection methods might have misidentified this atypical toe position during the swing phase as an early HS, leading to a higher measurement of paretic SLS. This rationale is supported by the fact that paretic SLS was the only overestimated parameter by the MMC in our study. Despite the fact that Lonini et al. [38] used an HPE algorithm customized for stroke survivors, the study reported identical correlations to our work for both paretic and non-paretic SLS. Other than post–stroke motor impairments, a possible reason for the low agreement in SLS parameters is the introduction of noise in the analysis. MMC has previously suffered from noise, possibly due to uplifting of the sampling frequency [47]. Thus, MMC detection of TO gait event was less accurate than HS [47], leading to a lower reliability in measuring temporal–based gait parameters that required estimation of TO, such as SLS [47]. Our study seems to be similarly affected by noise. While the KinaTrax MMC was downfiltered to 10 Hz sampling frequency, the IWS maintained a high frequency rate of 120 Hz. Therefore, the noise in detecting gait events might have originated from the IWS in our study [48]. Alternatively, the unmatched sampling frequencies between the two systems per se may have altered the sensitivity of detecting the TO gait event in our sample.

In general, our findings revealed subtle differences between CS and FS agreements between MMC and IWS. This small difference can be attributed to the limited gait adaptability of stroke survivors, which manifests in their inability to transition from comfortable to maximum gait speed. It has been shown that stroke survivors have significantly smaller capacity to modulate gait speed compared to individuals without stroke [49]. Post hoc, we further evaluated Gait Speed Reserve (GSR), a measure of gait adaptability that represents the difference between CS and FS. We found that the GSR in our sample was 0.4 m/s. This value corresponds to the limited GSR of stroke survivors reported in the literature compared to individuals without stroke [49]. Therefore, it appears that stroke survivors in our sample were unable to increase gait speed in a manner that would clearly demonstrate the change in spatiotemporal gait parameters between the two gait speed conditions, limiting the ability to show clear differences between MMC and IWS agreements at different gait speeds. Nonetheless, MMC and IWS agreements were generally higher during FS than CS for all gait parameters. Most notably, stance time (paretic and non-paretic) showed excellent agreement, and non–paretic SLS revealed good agreement. It is known that gait variability in stroke survivors is more pronounced during slower gait speeds than during faster gait speeds [50,51]. Thus, the accuracy of MMC measurements seems to be higher in FS conditions because of less gait variability.

We found that the bias (mean difference) between the MMC and IWS was small, and the LoAs were almost evenly distributed around zero for all spatiotemporal parameters. These findings indicate that the MMC and IWS essentially report the same average values without any systematic errors. In addition, the bias did not exceed Minimal Detectable Changes (MDC) reported in the literature for stroke survivors [19,52,53]. Some of the LoAs in our sample were large, suggesting that individual measurements might fall outside the MDC, namely DLS, stance time, SLS, and step length exceeded the associated MDC in both the paretic and non–paretic limbs [52,53]. However, most MDC values found in prior studies were recorded either using marker-based motion capture or during treadmill walking rather than overground walking [52,53]. Different methodological approaches may limit a direct comparison between our study results and other studies. Actually, a systematic review and meta–analysis that identified MDC of IWS–obtained spatiotemporal gait parameters [19] reported only two MDC parameters in stroke survivors, namely gait speed and stride length [19]. Our findings were well within these reported MDC values, suggesting that KinaTrax MMC can be used interchangeably with IWS in the clinic, with more caution when interpreting individual measurements of stance time, SLS, and step length.

Our study has several limitations. First, the majority of our study cohort were mildly impaired by stroke, which limits the generalizability of our findings to moderate or severe stroke survivors. Second, the KinaTrax MMC system was unable to identify some of the trials due to insufficient luminance in the background, or due to a similarity in coloring between the background and participant’s skin, clothing, and/or shoes. Additionally, the cameras have a limited field of view, reducing the number of gait cycles that could be paired with the IWS for inclusion. Thus, the KinaTrax MMC needs a minimum requirement of light, contrast in colors, and space, which constrains its neutral application. Notably, the presence of an assistive walking device (e.g., cane/walker) did not appear to influence the tracking of the KinaTrax model. Third, the absence of stroke survivors in the training history of the general human motion model used in the KinaTrax MMC could limit the accuracy of gait analysis, specifically in those with extreme gait asymmetries. Therefore, these results may vary with the version of the general human motion model, should population–specific training data be incorporated into the model. Fourth, we did not include a comparison between MMC and clinical outcome measures in a post-stroke population. These measures are important to improve the ability of MMC to infer functional independence within stroke survivors. Lastly, our results reflect a laboratory-based use of MMC. The influence of environmental distractions on the KinaTrax MMC validity is unknown.

## 5. Conclusions

In conclusion, the KinaTrax MMC is a valid gait analysis technology to measure spatiotemporal gait parameters in stroke survivors during comfortable and fastest walking speed conditions, particularly for gait speed, DLS, stride length, stance time, and step length. Measures of stride width and SLS should be interpreted with caution. Future studies are warranted to validate MMC with a more variable cohort of stroke survivors. Additionally, further work could also compare MMC with post–stroke clinical outcomes, like measures of motor impairments and functional limitations, and to validate MMC in unconstrained environments and thereby improve MMC clinical and ecological applications.

## Figures and Tables

**Table 1:** Participants’ demographics and stroke-related characteristics are shown. NIHSS: National Institute of Health Stroke Scale. FAC: Functional Ambulation Category. MoCA: Montreal Cognitive Assessment.

Total = 19	Median (IQR) or n (%)
Participants Demographics	
Age, years	63 (55–65)
Sex, male	10 (53%)
Race/Ethnicity	
*Non-Hispanic White*	4 (21%)
*Non-Hispanic Black*	7 (37%)
*Hispanic*	8 (42%)
**Stroke Characteristics**	
Time since stroke, months	33 (14–109)
Hemiparetic side, right	10 (53%)
NHISS	
NIHSS (0–5)	14 (74%)
NIHSS (6–15)	5 (26%)
FAC	
*FAC = 3*	2 (11%)
*FAC = 4*	8 (42%)
*FAC = 5*	9 (47%)
MoCA	23 (20–25)

**Table 2:** Comfortable speed agreement indicators (correlation correlations (r) and *p*-values, Intraclass Correlation Coefficients (ICCs), two-way mixed, single measure, and the 95%CI of absolute agreements and relative consistencies) between the markerless motion capture and the instrumented walkway are represented. DLS: double-limb support time. SLS: single-limb support time.

Parameter		Correlation Coefficient	Absolute Agreement	Relative Consistency
		r, *p*	ICC (95%CI)	ICC (95%CI)
**Two-limb**	Gait speed (m/s)	0.996, *p* < 0.001	0.999 (0.997–0.999)	0.998 (0.997–0.999)
	DLS (s)	0.768, *p* < 0.001	0.804 (0.684–0.881)	0.801 (0.680–0.880)
	Stride length (m)	0.997, *p* < 0.001	0.998 (0.997–0.999)	0.998 (0.997–0.999)
	Stride width (m)	0.554, *p* < 0.001	0.442 (0.198–0.634)	0.438 (0.195–0.630)
**Single-limb**	**Paretic**			
	Stance time (s)	0.858, *p* < 0.001	0.912 (0.854–0.948)	0.911 (0.852–0.948)
	SLS (s)	0.431, *p* < 0.001	0.314 (0.057–0.533)	0.317 (0.057–0.538)
	Step length (m)	0.973, *p* < 0.001	0.976 (0.959–0.986)	0.976 (0.959–0.986)
	**Non-Paretic**			
	Stance time (s)	0.867, *p* < 0.001	0.958 (0.928–0.975)	0.958 (0.928–0.975)
	SLS (s)	0.691, *p* < 0.001	0.731 (0.578–0.834)	0.734 (0.582–0.837)
	Step length (m)	0.969, *p* < 0.001	0.982 (0.968–0.989)	0.981 (0.968–0.989)

**Table 3:** Fastest speed agreement indicators (correlation correlations (r) and *p*-values, Intraclass Correlation Coefficients (ICCs), two-way mixed, single measure, and the 95%CI of absolute agreements and relative consistencies) between the markerless motion capture and the instrumented walkway are represented. DLS: double-limb support time. SLS: single-limb support time.

Parameter		Correlation Coefficient	Absolute Agreement	Relative Consistency
		r, *p*	ICC (95%CI)	ICC (95%CI)
**Two-limb**	Gait speed (m/s)	0.996, *p* < 0.001	0.998 (0.997–0.999)	0.998 (0.997–0.999)
	DLS (s)	0.843, *p* < 0.001	0.917 (0.859–0.952)	0.916 (0.857–0.951)
	Stride length (m)	0.997, *p* < 0.001	0.997 (0.995–0.998)	0.997 (0.995–0.998)
	Stride width (m)	0.508, *p* < 0.001	0.476 (0.230–0.663)	0.471 (0.227–0.659)
**Single-limb**	**Paretic**			
	Stance time (s)	0.928, *p* < 0.001	0.960 (0.931–0.977)	0.959 (0.929–0.976)
	SLS (s)	0.446, *p* < 0.001	0.480 (0.240–0.665)	0.481 (0.239–0.667)
	Step length (m)	0.985, *p* < 0.001	0.989 (0.981–0.994)	0.989 (0.980–0.994)
	**Non-Paretic**			
	Stance time (s)	0.942, *p* < 0.001	0.969 (0.944–0.983)	0.972 (0.951–0.984)
	SLS (s)	0.846, *p* < 0.001	0.886 (0.807–0.934)	0.890 (0.815–0.936)
	Step length (m)	0.977, *p* < 0.001	0.987 (0.978–0.993)	0.987 (0.977–0.993)

## Data Availability

The raw data supporting the conclusions of this article will be made available by the authors on request due to restrictions related to privacy.

## Abbreviations

The following abbreviations are used in this manuscript:

10MWT	10-Meter Walk Test
IWS	Instrumented Walkway System
MMC	Markerless Motion Capture
2D	2-Dimensional
3D	3-Dimensional
CNN	Convolutional Neural Network
CS	Comfortable Speed
FS	Fastest Speed
DLS	Double Limb Support
SLS	Single Limb Support
HPE	Human Pose Estimation
HS	Heel Strike
TO	Toe Off
GSR	Gait Speed Reserve
